# Sorbitol Reduces Sensitivity to *Alternaria* by Promoting Ceramide Kinases (*CERK*) Expression through Transcription Factor *Pswrky25* in Populus (*Populus simonii* Carr.)

**DOI:** 10.3390/genes13030405

**Published:** 2022-02-24

**Authors:** Meng Qi, Rui Wu, Zhihua Song, Biying Dong, Ting Chen, Mengying Wang, Hongyan Cao, Tingting Du, Shengjie Wang, Na Li, Qing Yang, Yujie Fu, Dong Meng

**Affiliations:** 1 The Key Laboratory for Silviculture and Conservation of Ministry of Education, Beijing Forestry University, Beijing 100083, China; qimqmm@163.com (M.Q.); wuruiv2001@163.com (R.W.); sxhxf0801@163.com (Z.S.); dongbiying1029@163.com (B.D.); chenting344@163.com (T.C.); wmy980102@163.com (M.W.); hongyan89945@163.com (H.C.); tingtingdu0827@163.com (T.D.); wangshengjiemail@126.com (S.W.); 13811302271@163.com (N.L.); yang.qing1020@163.com (Q.Y.); 2 The Institute of Tree Development and Gene Editing, Beijing Forestry University, Beijing 100083, China; 3 College of Chemistry, Chemical Engineering and Resource Utilization, Northeast Forestry University, Harbin 150040, China

**Keywords:** *Populus simonii* Carr., sorbitol feeding, disease resistance, *PsWRKY25*

## Abstract

Sugar, acting as a signal, can regulate the production of some chemical substance during plant defense responses. However, the molecular basis and regulatory mechanisms of sugar in poplar and other forest trees are still unclear. Sorbitol is a sugar-signaling molecule associated with plant defense. In this study, the pathogen-infested status of poplar was alleviated after exogenous feeding of 50 mM sorbitol. We sequenced and analyzed the transcriptome of poplar leaves before and after inoculation. The results showed that the genes *PR1*, *WRKY*, ceramide kinases (*CERK*) and so on responded to sorbitol feeding and pathogen infestation. We screened for genes related to disease resistance such as *PsWRKY25* and *PsCERK1* and found that significant disease spots occurred on day six of strep throat infestation. Under sorbitol feeding conditions, the appearance of spots was delayed after the pathogen inoculation. Due to the overexpression of *PsWRKY25*, the overexpression of *PsCERK1* triggered the defense response in poplar. This was also confirmed by *PsWRKY25* overexpression experiments. These findings present new insights into the influence of sorbitol on *Populus simonii* Carr. disease resistance. These results emphasize the value of molecular phenotypes in predicting physiological changes.

## 1. Introduction

Populus is an important greening and afforestation tree species, which plays an irreplaceable and huge role in wood production, urban greening and ecological construction [1]. The growth and development process are greatly affected by the surrounding environmental factors. Among them, the spread of various pathogens often causes large-scale tree diseases [2]. It is urgent to study the control methods of poplar diseases and the cultivation of improved varieties. At present, the main control methods of poplar diseases are pathogens [3], popular model and predictive control technology [4]. How sugar signals such as sorbitol can affect the susceptibility of poplar to pathogens at the genetic level is a new perspective in poplar disease resistance research.

Poplar leaf blight, also known as poplar leaf spot, is a common disease of poplar leaves caused by *Alternaria* [5]. It endangers the tender and young stems of the plant, resulting in slow growth and development of the tree. In serious cases, it will cause the whole plant to dry up and fall leaves prematurely, even cause the death of the tree. *Alternaria* has a wide range of species and hosts, including *Ginkgo biloba*, *Metasequoia glyptostroboides*, *Paeonia suffruticosa*, *Malus domestica*, *Pyrus* spp., *Cerasus pseudocerasus*, *Citrus reticulata*, *Juglans regia* and other woody trees, fruit trees, crops, Oil crops, cruciferous plants and model plant *Arabidopsis thaliana.* They will not only cause the most common leaf spot diseases but also fruit mildew and rot [6,7,8]. The hazards of *Alternaria* to plants are sufficient to explain and it is one of the main groups causing plant pathogen diseases [9]. Many researchers [10] have also proved that poplar can respond to the infection of *Alternaria*. Therefore, the study of the impact of *Alternaria* on poplar is of great significance for the study of poplar disease resistance. 

Sorbitol is a typical sugar alcohol compound. Sugar alcohols are the main end products in photosynthesis and carbohydrate transport in many plants [11,12]. Sorbitol is mainly produced in the fruits of Rosaceae species such as apple (*Malus domestica*). Sorbitol accounts for 60–80% of the photosynthetic products produced in leaves and transported in phloem [13]. Sorbitol is synthesized in the cytoplasm of source leaves through a two-step process in plants. In plants, D-glucopyranose 6-phosphate is converted to sorbitol 6-phosphate by aldose 6-phosphate reductase (*A6PR*), then sorbitol 6-phosphate is dephosphorylated to sorbitol by sorbitol 6-phosphate phosphatase [14]. During the growth and development, sorbitol is usually used as a signal molecule, which is mainly metabolized into fructose through sorbitol dehydrogenase to support plant growth and development. In related studies by Dong Meng et al., sorbitol also acts as a signal molecule to regulate glucose metabolism, stamen development and pollen tube growth [15]. The previous research of Prof. Lailiang Cheng’s Cornell Group also found that sorbitol can regulate Apple disease resistance by regulating *NLR* family genes [16]. However, it is still not clear whether sorbitol can act as a signal molecule to affect the expression of specific genes in poplar, so as to regulate the immune response of poplars to pathogen infection. 

In the present study, we propose an application exogenous sorbitol to *Populus simonii* Carr.. By GO and KEGG analysis of transcriptome sequencing data, comparing pathogenic leaves sorbitol and water feeding, the RNA transcript levels of infected leaves were analyzed based on approximately 2660 differentially expressed genes (DEGs). We analyzed how *PsWRKY25* and *PsCERK1* regulate internal changes in poplar under the influence of sorbitol signaling to resist pathogen infestation. The analysis showed that defense-related transcripts were increased in sorbitol feeding in comparison to the water feeding. We think that leaves with sorbitol feeding were more resistant to pathogen infection. We verified by overexpression experiments that exogenous sorbitol could up-regulate the expression of *PsWRKY25*, which was followed by a significant increase in the expression of *PsCERK1*. These findings provide new insights into the research of sorbitol on disease resistance in forest trees.

## 2. Materials and Methods

### 2.1. Plant Material

The *Populus simonii* Carr. leaves come from the Beijing Forestry University Technology Greenhouse. *Alternaria* was obtained from field-collected poplar leaves showing black spots on the leaves. We cloned the ITS sequence of *Alternaria* and sequenced it to verify the pathogenic species. 

### 2.2. Feeding Leaves with Exogenous Sorbitol

*Populus simonii* Carr. leaves from the laboratory of Beilin science and technology center were fed for 6 h. The control group with water feeding and processing group with sorbitol feeding at 50 mmol/L (50 mM). After feeding, the inoculation experiment was carried out. The status after inoculation was observed. The sorbitol feeding experiment was performed by feeding plant leaves through the transpiration stream. The leaf samples of 0 dpi (days post-inoculation), 1 dpi, 2 dpi and 3 dpi *Populus simonii* Carr. after inoculation were collected, respectively. This method comes from the article of Meng et al. [16]. The youngest fully expanded leaves were cut from the twigs with scissors, floated on the water, then randomly assigned to 50 mM sorbitol or water control. Each treatment was repeated three times, with 12 leaves each time. In each culture dish, only the petiole is sandwiched between the water and the bottom of the glass dish to completely soak it in the liquid. Put the filter paper (round) soaked in water and sorbitol in a plastic dish. After feeding, the leaves were inoculated with the pathogen, then the leaves were incubated at 23 °C in the dark, finally the leaves were sampled and photographed according to the time designed in the materials and methods. After the samples were collected, the *Populus simonii* Carr. leaf samples were frozen in liquid nitrogen and stored at −80 °C for performing RNA extraction experiments. Lesion size analysis was carried out for the photos taken. 

### 2.3. Isolation, Purification, Identification and Inoculation of Alternaria

**Figure 1:**
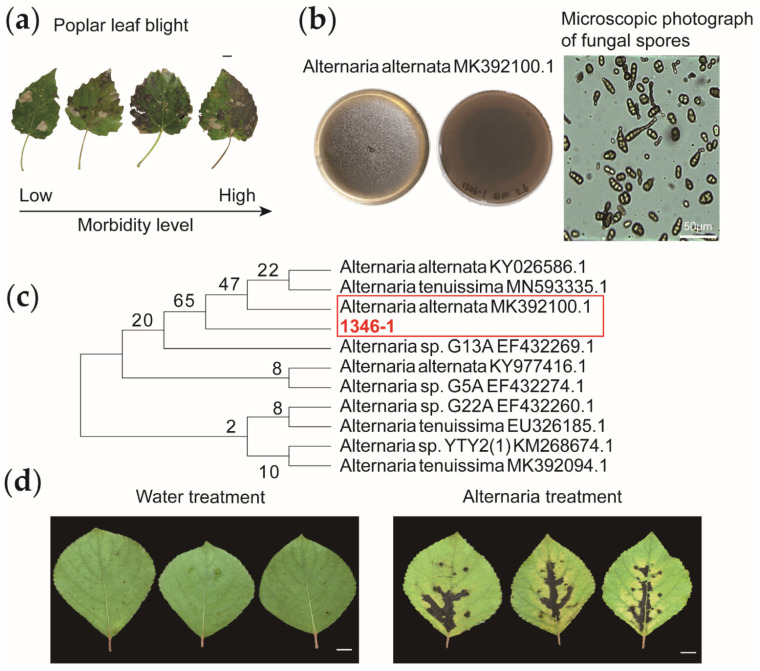
Phenotype, treatment and identification methods of infected leaves. (**a**) Poplar diseases cause leaf spot-like symptoms on poplar leaves. (**b**) A Petri dish for pathogens and microscopic photograph of the *Alternaria*. (**c**) It indicates the identification results of pathogen. *Alternaria alternata* (KY026586) is 599 bp; *Alternaria alternata* (KY977416) is 594 bp; *Alternaria alternata* (MK392100) is 599 bp; *Alternaria* sp. *G5A* (EF432274) is 738 bp; *Alternaria* sp. *G13A* (EF432269) is 707 bp; *Alternaria* sp. *G22A* (EF432260) is 735 bp; *Alternaria* sp. *YTY2(1)* (KM268674) is 597 bp; *Alternaria tenuissima* (EU326185) is 596 bp; *Alternaria tenuissima* (MK392094) is 596 bp; *Alternaria tenuissima* (MN593335) is 599 bp. The red box represents the pathogen identification result. (**d**) It was water-treated and *Alternaria* pathogen-treated *Populus simonii* Carr. leaves, respectively. Bars, 1 cm.

The leaves collected in the field were sampled to isolate pathogenic microorganisms. We selected leaves with black spot-like lesions on the leaf parts for follow-up studies. Using the Koch postulates, firstly the leaves were disinfected with 75% ethanol, sodium hypochlorite and sterile water, and then the diseased and healthy connection parts were cut into small pieces about 5 mm × 5 mm with sterilized scissors. Finally, these small pieces were inoculated on a PDA medium for culture. After the plaque grows, inoculate the plaque on a new PDA plate group for culture until uniform and uniform colonies grow on the plate. The spores in the figure spread from the center to the periphery (Figure 1b). We used pathogens cultured for 20 to 30 days. After the pathogen grew all over the plate, the pathogen spores were scraped off with a glass rod and ground with liquid nitrogen. The subsequent experiments such as DNA extraction and pathogen genus identification were carried out. The DNA of the fungus was extracted by the CTAB method. We cloned the ITS sequence of the fungus’ DNA by PCR. The method we used was Internal Transcribed Spacer Identification. We compared the ITS sequence to determine the species of the fungus in NCBI. The remaining spores were dissolved in autoclaved double-distilled water and used for subsequent inoculation experiments. Following DNA sequencing (Zhongmei Taihe Biotechnology Co., Beijing, China), NCBI BLAST was performed to identify that the isolated pathogen belongs to *Alternaria alternata*. The pathogen plates identified in the above experiments were diluted to form pathogen solution. The spore number of the pathogen solution was observed under the microscope. The spore solution used was 2.5 × 10^6^ spores per ml. The pathogen solution diluted to the appropriate spore number was inoculated on the *Populus simonii* Carr. leaves. Small wounds were created on the leaves with a needle before inoculation, followed by inoculation of the spore solution onto the leaves. For each replicate we selected 12 leaves of uniform size. Each sample had three biological replicates.

### 2.4. RNA Extraction and Transcriptome Sequencing

There are 12 samples in total, each containing 12 *Populus simonii* Carr. leaves. These samples include 4 sampling times. Each sampling point had three biological replicates. These sampling points were after sorbitol or water feeding and after pathogen inoculation. Ribonucleic acids were extracted from 12 samples using tri ZOL (Thermo Fisher Scientific Inc., Waltham, MA, USA) [17,18]. All RNAs were classified using Illumina Nova SEQ platform 6000 (Illumina Inc., San Diego, CA, USA). NanoPhotometer spectrophotometer was used to evaluate the RNA quality. The error rate is less than 0.03%. A total of 83.538 Gb of clean data (post-quality control sequencing data) was obtained from the transcriptome analysis of 12 samples. 

### 2.5. Analysis of Differential Expression, Transcriptome Alignment and Functional Enrichment

Total RNA was extracted from treated and control leaves followed by transcriptome sequencing (Yuanxin Biomedical Technology Co., Shanghai, China). Gene rpkm deviation within 15% of final value. The reads with an error rate greater than 0.1 were filtered out. We filtered out readings containing more than 10% ambiguous bases. We also filtered out readings less than 50 bp in length. The remaining high-quality readings are consistent with the latest *Populus simonii* Carr. genome. The percentage of Q30 bases was above 91.63% and the GC content ranged from 43.97% to 45.69%. HISAT2 was used to align obtained reads to the reference genome. DEGs are tested by accurate testing and estimated dispersion. Software: DeSeq2, parameters: differential expression of genes with FDR < 0.05, log2 (Fold Change) ≥ 1, log2 (Fold Change) ≤ −1 and Q value < 0.05. DEGs are designated as “up-regulated” and “down-regulated” according to their expression level above or below the untreated state. Heat maps are drawn using Log_2_(FC) or FPKM values for all DEG’s. The heat map is drawn by Microsoft Office Excel. To classify genes into functional categories, protein sequences were compared with gene databases for GO and KEGG enrichment. All DEGs were involved in GO and KEGG concentration analysis. The phyper function in R software was used for GO and KEGG enrichment analysis. We took *Populus tomentosa* genome as the reference genome. GO and KEGG pathway with P-value lower than 0.05 was significantly enriched.

### 2.6. Analysis of Transcription Factor Binding Site in the Promoter Region

To study the transcription factor binding sites in the promoter region related to disease resistance and immunity, the genomic DNA sequence about 2000 bp upstream of the start codon was searched. NCBI (https://www.ncbi.nlm.nih.gov, accessed on 21 January 2022) was used to find DNA sequences. Plant PAN (http://plantpan.itps.ncku.edu.tw, accessed on 28 September 2021) was used to predict transcription factors. TB tools software (https://github.com/CJ-Chen/TBtools, accessed on 4 October 2021) and other software were used to analyze the binding sites of the promoter region. The TB tools software was also used to visualize the promoter region. 

### 2.7. Construction of Transgenic Vector

To overexpress *PsWRKY25* in *Populus simonii* Carr., we ligated the coding sequence of *PsWRKY25* to the pROKII vector to generate the overexpression vector pROKII-*PsWRKY25*-GFP-OE. The vector primer sequences are shown in Supplementary Table S3. Firstly, specific primers were designed by primer 5.0 software. Es Taq (Kangwei Century Bio, Jiangsu, China) was used to perform PCR experiments. *PsWRKY25* was cloned from *Populus simonii* Carr.’s cDNA by PCR and subsequently it was sequenced to determine the sequence information of *PsWRKY25*. After colony PCR identification of the growing plaque, the *PsWRKY25* coding sequence was ligated to pROKII. Finally, the gene was transformed to the *Escherichia coli* DH5α cells. Recombinant plasmids were transformed into *Agrobacterium* strain GV3101 in bacterial culture medium (YEP liquid medium supplemented 25 mg·L^−1^ rifampin and 50·mg L^−1^ kanamycin), the culture was shaken on a rocking platform at 180 rpm at 28 °C for ~12 h.

### 2.8. Transient Transformation System of Populus simonii Carr

The leaves of *Populus simonii* Carr. aseptically cultured seedlings were taken for transient transfection experiment [19,20]. A transient transfection experiment was performed by immersing *Populus simonii* Carr. leaves in *Agrobacterium* GV3101 and the gene was overexpressed on *Populus simonii* Carr. leaves by using the *Agrobacterium* vacuum infiltration method [21]. After the *PsWRKY25* was overexpressed, the leaves were inoculated with the *Alternaria alternata*. Collect samples from *Populus simonii* Carr. leaves at 0 dpi, 1 dpi, 2 dpi and 3 dpi. The samples are sampled with liquid nitrogen and stored at −80 °C for subsequent experiments.

### 2.9. RNA Extraction and Real-Time Quantitative PCR (RT−qPCR) Validation

RNA extraction kit (Thermo Fisher Scientific Inc., MA, USA) was used to extract the total RNA of the sample. We used agarose gel electrophoresis to detect the degree of RNA degradation and integrity, and NanoDrop 8000 (Thermo Fisher Scientific, Waltham, Massachusetts, USA) to detect the degree of RNA contamination. The reverse transcribed double-stranded cDNA was then obtained using the rapid quantitative RT Supermix Kit (M-MLV Reverse Transcriptase, Mi Si Century Biotechnology company, Shanghai, China). Using the leaf cDNA of *Populus simonii* Carr. as the template, the specific primers and fluorescence quantitative PCR primers were designed by primer 5.0 software. The expression was verified again by real-time fluorescence RT−qPCR. RT−qPCR used a rapid polymerase chain reaction premix (SYBR Green, Kang Wei Century Biotechnology company, Jiangsu, China) for three biologically repeated different cDNA at CFX Connect Thermal Cycler (Bio-Rad Laboratories). The internal control was *UBQ* (LOC118044514) according to our previous studies, which was widely used in *Populus tomentosa*. Primers for these genes were listed in Supplementary Table S2. PCR reaction system: cDNA 1 µL, 10 µmol/L upstream and downstream primers 0.3 µL each, SYBR Green Master mix (2×) 5 µL, ddH2O 3.4 µL. PCR reaction procedure: pre-denaturation at 95 °C for 30 s; 95 °C for 5 s, 60 °C for 30 s, 40 cycles. The relative expression of genes was assessed according to the 2^−^^△△CT^ method [22]. We analyzed RT−qPCR results by using Microsoft Office Excel. Each sample had at least three biological replicates. 

### 2.10. Statistical Analysis

The data were analyzed by one-way analysis of student *t*-test. The “*” and “a”, “b”, “c”, “d” different letters indicate significant difference. There was a significant difference between the control group and the processing group (*p* < 0.05). All the charts were drawn with GraphPad Prism 9. These graphs also used MEGA7, Adobe Illustrator CS6 and Adobe Photoshop CS6.

## 3. Results

### 3.1. Sorbitol Feeding Improves Populus simonii Carr Resistance to Alternaria

Phylogenetic tree analysis revealed that the pathogen belongs to *Alternaria* (Figure 1c). *Alternaria* spreads in the wild causing large black spots on poplar leaves resulting in leaf wilt and death (Figure 1a). *Alternaria* was inoculated on the healthy *Populus simonii* Carr. leaves. It was found that the inoculated *Populus simonii* Carr. leaves would show the dark brown to black disease spots as shown in Figure 1 within 7–14 days, the diseased spots infested by the pathogen would gradually expand with the extension of infection time (Figure 1d). To study the effect of internal feeding of sorbitol on the disease resistance of *Populus simonii* Carr., we carried out feeding experiment before inoculation. After 6 h of feeding, *Populus simonii* Carr. immediately carried out pathogen inoculation experiment. 

**Figure 2:**
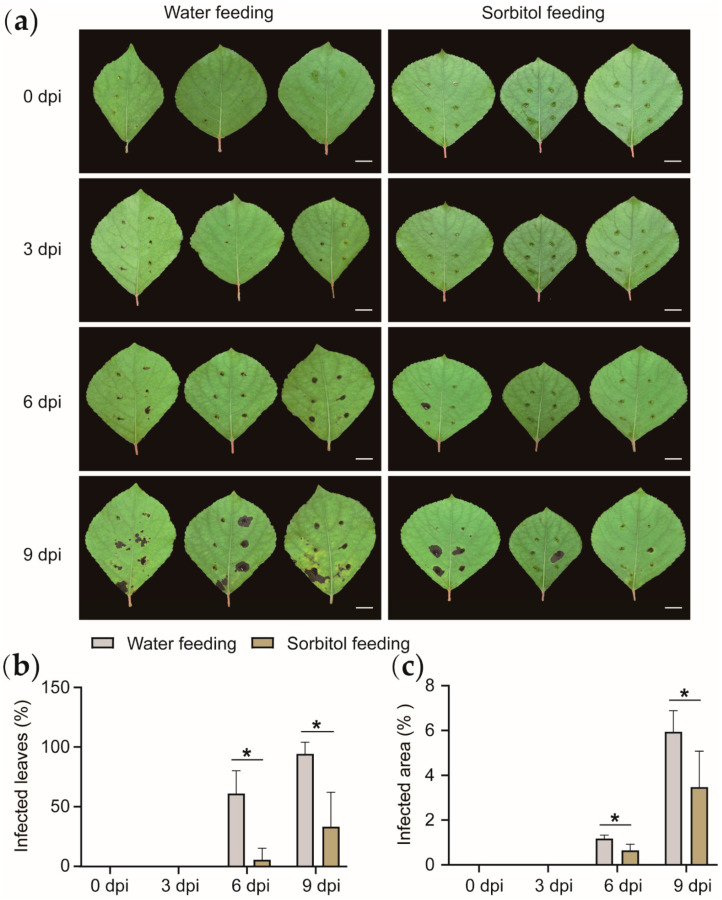
Statistics of leaf phenotype and physiological indexes of *Populus simonii* Carr. after feeding and pathogen infestation. (**a**) It indicates the leaf state of *Populus simonii* Carr. 0, 3, 6 and 9 days after infestation. Bars, 1 cm. (**b**) It indicates the statistics of infection rate of *Populus simonii* Carr. leaves in different treatments and different periods. (**c**) It indicates the proportions of infected area of *Populus simonii* Carr. leaves in different treatments and different periods. (* stands for *p* < 0.05, with significant difference.)

The *Populus simonii* Carr. leaves with sorbitol feeding and the *Populus simonii* Carr. leaves with water feeding showed obvious differences after inoculation. As shown in (Figure 2a), the lesion area proportion of sorbitol feeding leaves after the same period of culture is smaller than that of water feeding leaves, the number of susceptible leaves was less than that of water feeding leaves (Figure 2b,c). We speculate that sorbitol feeding can partially increase the ability of leaves to resist *Alternaria*. Based on speculation, we will further explore the link between sorbitol and disease resistance.

### 3.2. Transcriptome Sequencing Reveals Significant Differences between Sorbitol-Feeding and Water Feeding

To further prove how sorbitol feeding affects leaf disease resistance, we sequenced the transcriptome of *Populus simonii* Carr. leaves after feeding and inoculation, using four samples, and constructed 12 sequencing libraries. Among them, the four samples include W, WI, S and SI. W represents water feeding; WI represents water feeding then infestation; S represents sorbitol feeding; SI represents sorbitol feeding then infestation. The sequencing results showed that, in the analysis of differential expressed gene results, SI and S have 2660 DEGs (the up-regulated genes 1218 and down-regulated genes 1442 are more than other genes), followed by WI and W with 1843 DEGs (884 up-regulated genes and 959 down-regulated genes), and SI and WI with 365 DEGs (344 up-regulated genes and 21 down-regulated genes). By comparison, S and W have relatively few DEGs, with 450 DEGs (425 up-regulated genes and 25 down-regulated genes) (Supplementary Figure S1). Continuing to analyze the gene function annotation, it was found that the GO, KEGG, NR, PFAM, STRING and SwissProt databases can contain the function annotation of all genes (Supplementary Figure S1). The total number of clear readings obtained from the cDNA library of each sequencing library shows that the gene abundance detected in our samples is sufficient, the length of transcripts produced in the experiment is also sufficient. In the sequence library, we obtained 83.538 GB of clean data. The average amount of clean data of each sample was 6.962 GB, the percentage of Q30 base was more than 91.63%, the GC content was between 43.97% and 45.69% (Supplementary Table S1). The comparison rate ranged from 86.665% to 88.012%. Further principal component analysis (PCA) using DESeq2 showed that there were very different expression profiles among SI, S, WI and W (Supplementary Figure S2). The expression profiles among repeated samples were very similar, but the expression profiles among *Populus simonii* Carr. treated with different treatments were completely different (Supplementary Figure S3). Of course, there were individual differences among samples, but it also showed that sorbitol feeding had a great effect on leaves.

**Figure 3:**
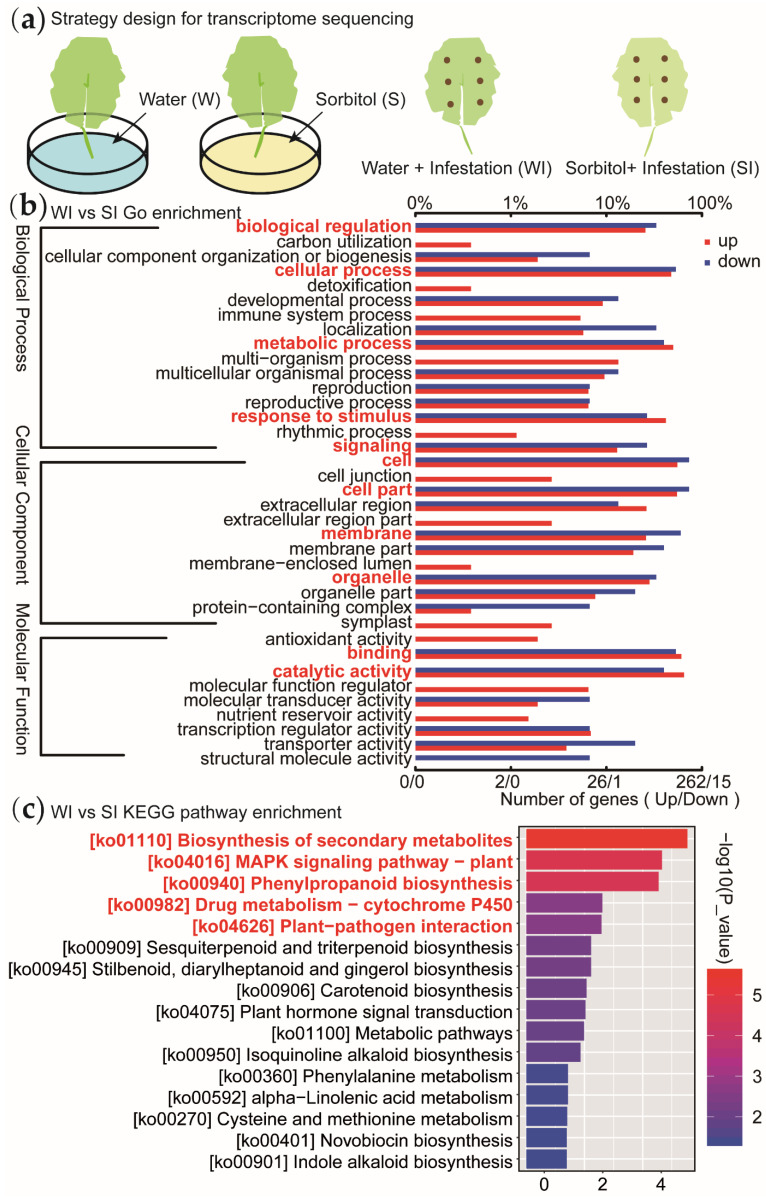
Strategy design and data analysis for transcriptome sequencing. (**a**) Strategy design for transcriptome sequencing. (**b**) GO enrichment analysis of differential expressed genes between sorbitol feeding and water feeding. (**c**) KEGG enrichment analysis of differential expressed gene between sorbitol feeding and water feeding. (W represents water feeding; WI represents water feeding then infestation; S represents sorbitol feeding; SI represents sorbitol feeding then infestation.)

Before sequencing, we first treated *Populus simonii* Carr. with sorbitol feeding and water feeding, followed by inoculation of its leaves with *Alternaria* pathogen, then took the leaves before and after pathogen inoculation as samples for transcriptome sequencing analysis (Figure 3a). Gene Ontology (GO) analysis showed that DEGs were in cell, cell part, binding, catalytic activity, Cellular process and other functional categories are obviously enriched (Figure 3b; Supplementary Table S4). Kyoto Encyclopedia of Genes and Genes (KEGG) notes show that the pathways rich in these DEGs are closely related to ko04626: plant pathogen interaction, ko04016: MAPK signal pathway (Figure 3c; Supplementary Table S5).

**Figure 4:**
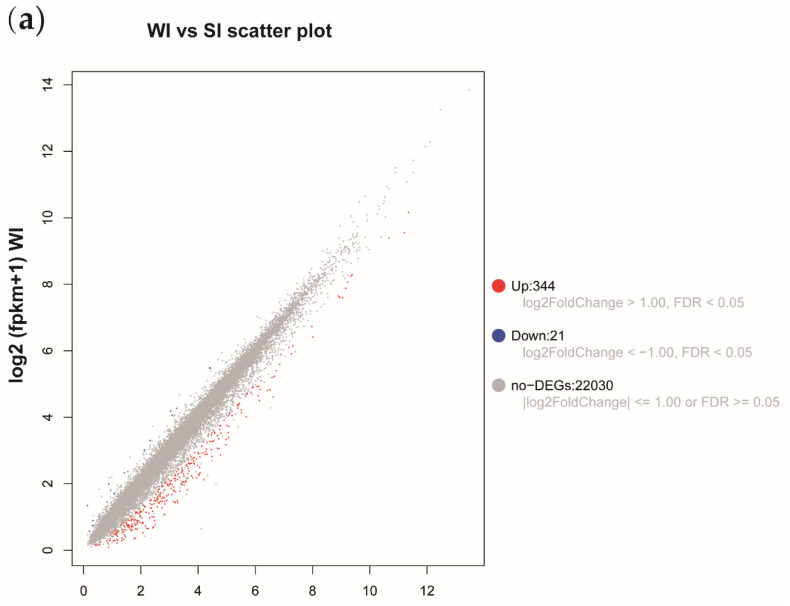

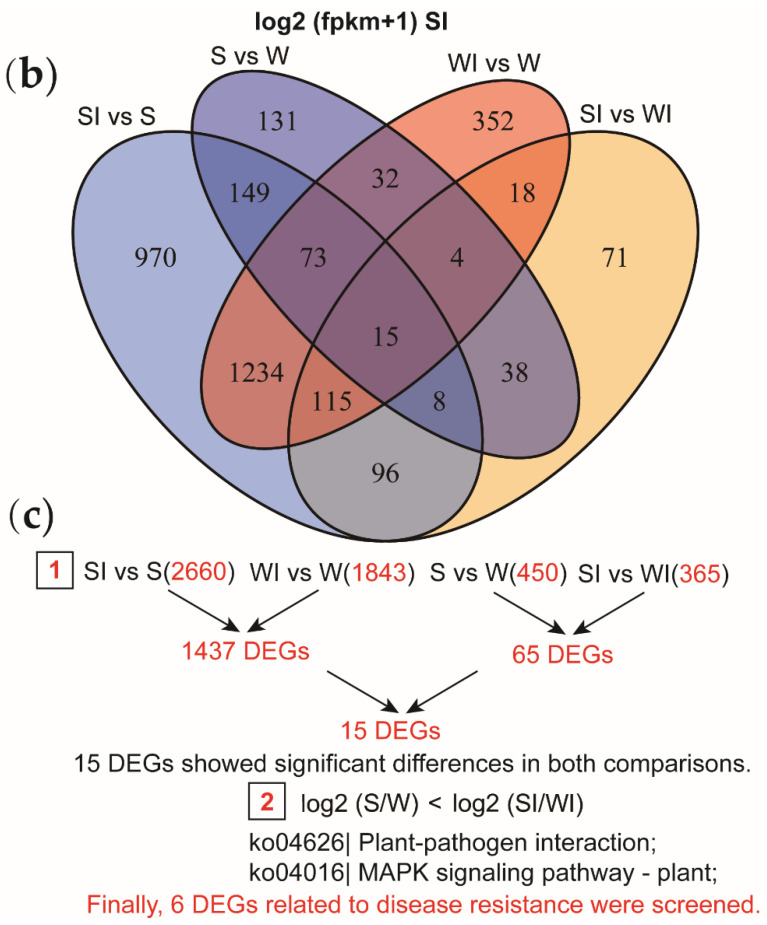
Differentially expressed genes (DEGs) screening strategy. (**a**) It indicates the difference genes scatter plots between WI and SI. (**b**) Venn diagram representing all differential genomes. (**c**) DEGs screening strategy. (W represents water feeding; WI represents water feeding then infestation; S represents sorbitol feeding; SI represents sorbitol feeding then infestation.)

Moreover, relevant studies in the field of botany reveal that most studies on plant disease resistance had focused on the two pathways of MAPK signal pathway and plant pathogen interaction. Therefore, our study also focused on the analysis of these two pathways. In the Venn, we were able to see that there were 2660 differential expressed genes with significant differences (Figure 4b; Supplementary Table S6). Comprehensive analysis shows that there were more common DEGs in the up-regulated DEGs (Figure 4a) Venn map than in the down-regulated DEGs Venn map, indicating that there are more up-regulated DEGs and more common up-regulated DEGs in the sequencing library. We think that sorbitol feeding can cause more gene up-regulation than water feeding. Among the 2660 differential expressed genes found, 1437 DEGs showed significant differences between inoculated and non-inoculated, and 65 DEGs showed significant differences between sorbitol feeding and water feeding, of which 15 DEGs showed significant differences in both comparisons. Finally, six coding genes related to plant disease resistance pathways were screened from the above DEGs (Figure 4c; Supplementary Table S8). The next research will mainly focus on the up-regulated differential expressed genes in different populations, analyze the effects of sorbitol on these up-regulated differential expressed genes and disease resistance, then analyze the role of sorbitol in improving the immunity of *Populus simonii* Carr..

### 3.3. Screening and Validation of Relevant Functional Genes in Disease-Resistance Related Pathways

**Figure 5:**
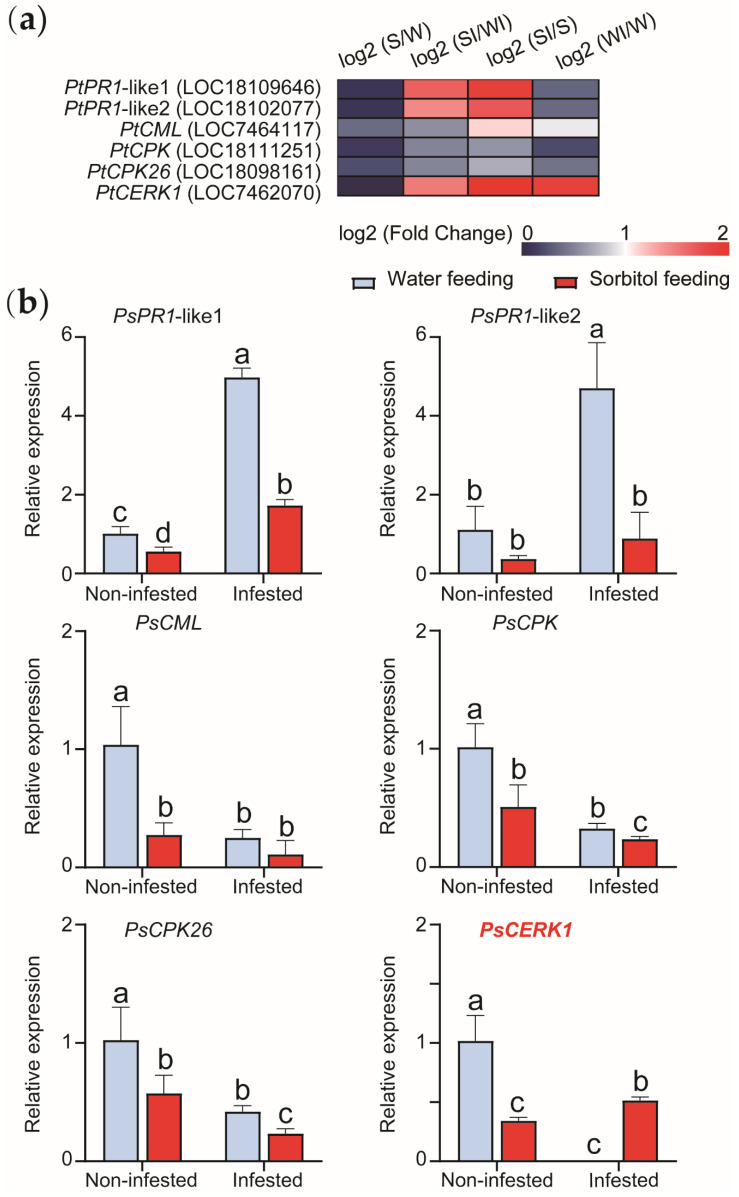
Functional gene screening. (**a**) Six genes were obtained and heat maps were drawn from log2 (Fold Change). (**b**) The expressions of six genes at different stages were analyzed by RT−qPCR. (W represents water feeding; WI represents water feeding then infestation; S represents sorbitol feeding; SI represents sorbitol feeding then infestation. Different lowercase letters indicate significant differences (*p* < 0.05).)

Selection from KEGG pathways such as plant disease resistance and immunity. Six functional genes with different expression at different culture stages were screened, which were *PtPR1*-like1 (LOC18109646), *PtPR1*-like2 (LOC18102077), *PtCML* (LOC7464117), *PtCPK* (LOC18111251), *PtCPK26* (LOC18098161), *PtCERK1* (LOC7462070) (Figure 5a). RT−qPCR was performed for six genes (Figure 5b). We found that the expression of one gene was higher after sorbitol feeding and inoculation than after water feeding. This result demonstrated that the gene *PsCERK1* responded to the sorbitol feeding and the pathogens infestation. The changes of disease resistance genes in leaves after sorbitol feeding were significantly different before and after inoculation. In contrast, the gene expression of leaves after water feeding decreased after inoculation.

### 3.4. Screening for Disease-Resistance-Associated Transcription Factors by Promoter Analysis

**Figure 6:**
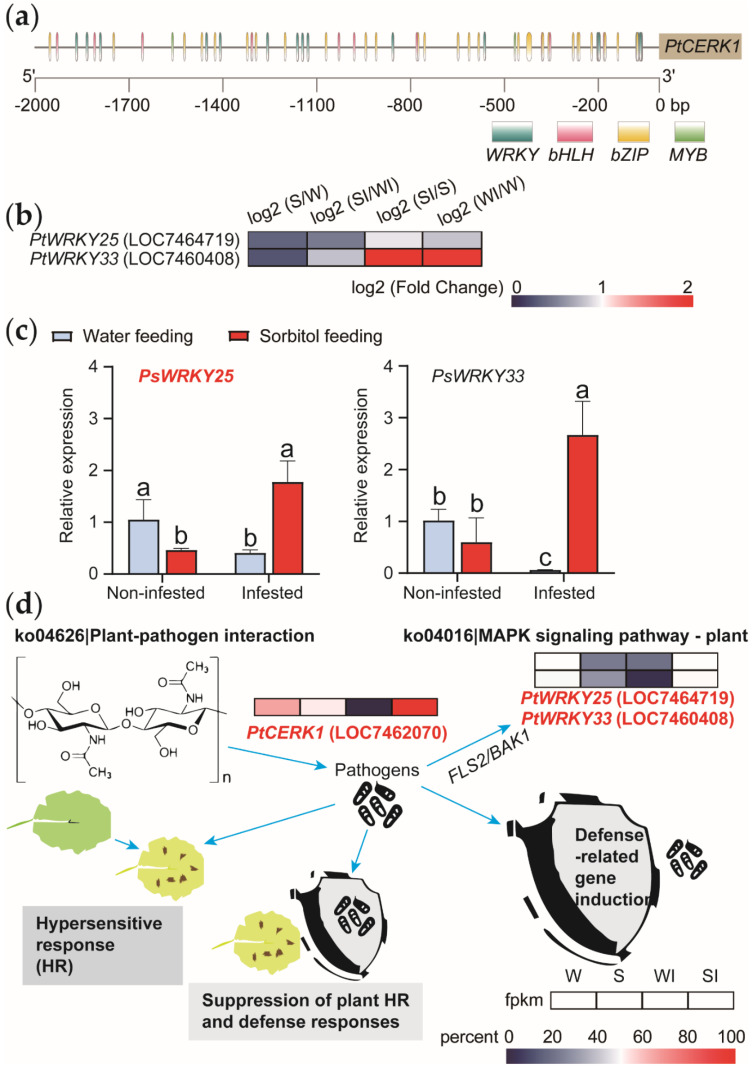
Analysis of promoter cis-elements and functional analysis of genes. (**a**) Prediction and analysis of transcription factors in gene promoter region. (**b**) Transcription factor heat map analysis. (**c**) The relative expression of two transcription factors by RT−qPCR. (**d**) Heat map analysis of disease resistance related KEGG pathway genes.

To continue to explore the regulatory mechanism of the gene screened above, we analyzed the disease resistance pathways (Figure 6b). *PtCERK1*, as a functional gene, affects the process of pathogen, defense-related gene induction, hypersensitive response (HR), suppression of plant HR and defense responses. Pathogen infection affected the expression of the *PtWRKY25* and *PtWRKY33*. Through the analysis of 2000 bp homeopathic elements upstream of the related gene in disease resistance, it is found that there are more *WRKY* binding sites in *PtCERK1* (Figure 6a, Supplementary Table S7). A large number of studies have also shown that *WRKY* family transcription factors have a key impact on plant disease resistance [23]. In the transcriptome data, we also analyzed these two genes, *PtWRKY25* and *PtWRKY33*, which showed differential expression under different treatments. We made the heat map of *PtWRKY25* (LOC7464719) and *PtWRKY33* (LOC7460408) (Figure 6b). Through RT−qPCR, we also measured the expression of the two transcription factors under different treatments (Figure 6c). Because the relative expression of *PsWRKY25* differed more significantly between different periods, it responds to both sorbitol and *Alternaria*. Therefore, we predict that the *PtWRKY25* can regulate the gene expression of *PtCERK1* in response to sorbitol signal in *Populus simonii* Carr. plants, thus affecting the realization of *Populus simonii* Carr. disease resistance and other functions. We made the heat map of these genes on the KEGG pathway (Figure 6d). Which can more intuitively indicate the different FPKM values of different genes under different treatments. We also identified the location of these key genes in the disease resistance pathway from the studies of others, found that their expression channels are in line with our prediction of the effect of sorbitol. Therefore, the next research plans to construct transgenic lines for these three genes to further clarify their functions, strive to further explain how sorbitol, as a signal substance, affects the disease resistance function and immune ability of *Populus simonii* Carr.

### 3.5. Overexpression of PsWRKY25 Increases the Expression of PsCERK1

**Figure 7:**
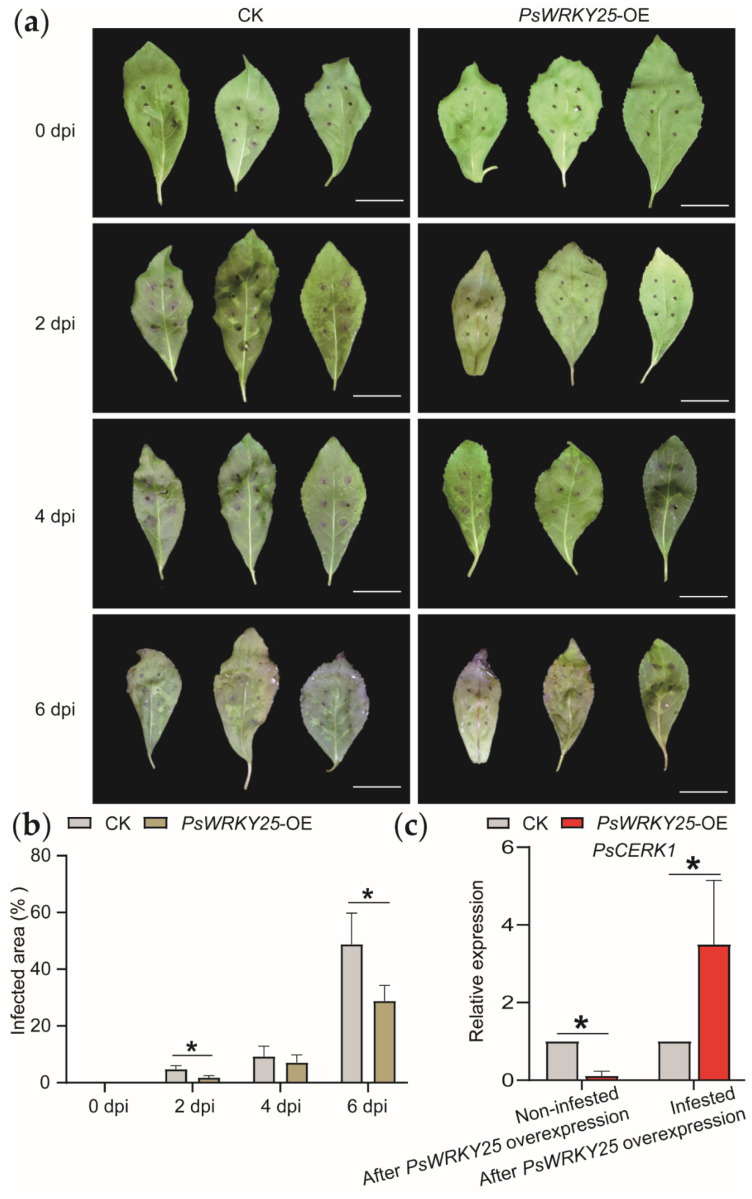
Overexpression of *PsWRKY25* up-regulates *PsCERK1* gene expression and improves disease resistance in poplar. (**a**) Leaf state of CK and *PsWRKY25*-OE. CK stands for transient transformation using eGFP-pROKII empty vector and *PsWRKY25*-OE (overexpression) stands for transient transformation using the overexpression vector pROKII-*PsWRKY25*-GFP-OE. Bars, 1 cm. (**b**) It indicates the proportions of infected area of *Populus simonii* Carr. leaves in CK, *PsWRKY25*-OE and different periods. (**c**) The relative expression of *PsCERK1*. It was measured in leaves during different periods of pathogen infestation. (* stands for *p* < 0.05, with significant difference.).

We chose to overexpress *PsWRKY25* because of the greater number of binding sites for the *WRKY* transcription factor on *PsCERK1* and more significant differences in gene expression between sorbitol feeding and water feeding. The function of *PsWRKY25* was further characterized by overexpression of the *PsWRKY25* on the leaves of young, tender *Populus simonii* Carr. histiocytic seedlings via transient infestation (Figure 7a). The gene function was verified by RT−qPCR in both treated leaves. We subjected CK (control check) and overexpression plants to pathogen infestation experiments at the same time. On 2 dpi, CK leaf spots began to appear (Figure 7a). On 6 dpi, basically all CK leaves showed lesions and spreading to the whole leaf. We then measured the proportions of the infected area of *Populus simonii* Carr. leaves in CK, *PsWRKY25*-OE and different periods. *PsWRKY25* overexpressing plants produced smaller areas of disease spots compared to CK at each infection stage. We also found that with the increase in infestation time, the disease spot area gradually expanded (Figure 7b). When *PsWRKY25* was overexpressed, the expression of *PsCERK1* in overexpressed leaves showed an increasing trend with increasing culture time. Moreover, the expression of *PsCERK1* in the *PsWRKY25* line showed a burst increase trend after inoculation with the pathogen. The expression of *PsWRKY25* in the *PsWRKY25* line also showed a burst increase trend after inoculation with the pathogen (Figure 7c, Supplementary Figure S4). These results suggest that the expression of *PsWRKY25* up-regulated the expression of *PsCERK1*, making plants less susceptible to pathogens. Based on all the results, we speculate that sorbitol does play an important role in poplar disease resistance. As a signal substance, sorbitol affects the content of *PsWRKY25* in *Populus simonii* Carr., then affects the content of *PsCERK1*. It increases the disease resistance of poplar.

### 3.6. Disease Resistance Mechanism of Poplar

**Figure 8:**
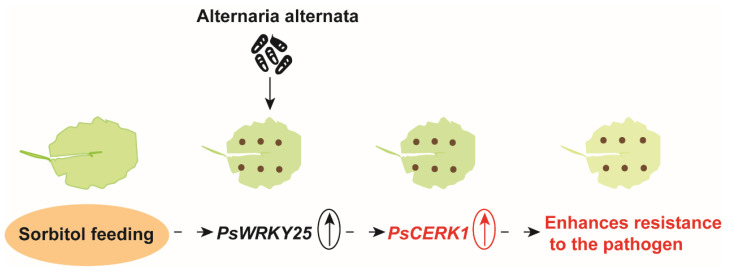
Mechanism analysis of sorbitol affecting plant disease resistance. Sorbitol treatment increased the expression of *PsWRKY25*, and the accumulation of *PsWRKY25* increased the expression of *PsCERK1*, which in turn indirectly or directly affected the susceptibility of *Populus simonii* Carr. to *Alternaria* for disease resistance purposes.

In the overexpressed leaves of *PsWRKY25*, the expression of *PsCERK1* increased. There are *WRKY* transcription factor binding sites on *CERK1*. We think that *PsWRKY25* will affect *PsCERK1*, when one side is overexpressed, the expression of the other side will be increased. They may simultaneously affect the susceptibility of *Populus simonii* Carr. to *Alternaria*, showed delayed production of leaf spots (Figure 8).

## 4. Discussion

The preliminary research of the group proved that sorbitol, as a kind of sugar substance commonly found in Rosaceae, has the effect of improving the resistance of plants to *Alternaria* [16]. Sorbitol is increasingly being studied as an emerging sugar alcohol signal substance [23]. It is important, not only for various aspects of human diseases [24], but also plant growth and development. Based on these views, the effect of exogenously applied sorbitol on the resistance of poplar leaf blight was of great significance to be researched. We explored the effect of sorbitol as a sugar signal on disease resistance in *Populus simonii* Carr., explored the molecular mechanism of disease resistance in *Populus simonii* Carr. with *WRKY* family genes as the entry point. We showed that sorbitol regulates resistance to *Alternaria* by regulating the expression of *PsWRKY25*. CK Populus minor leaves infested with the pathogen exhibited a normal diseased state with significant changes in the lesion on 6 dpi. *PsWRKY25*-OE infested with the pathogen exhibits a lesion-delayed growth phenotype, suggesting the role of *PsWRKY25* in poplar resistance to *Alternaria*. Sorbitol feeding could increase the expression of the *PsWRKY25* gene in poplar. This indicates that sorbitol can affect poplar disease resistance. Thus, our study investigated the effect of sorbitol on poplar using apple species of Rosaceae as a continuation [16], revealing that sorbitol can act as a signal to regulate the expression of relevant disease resistance genes in plants by differences in transcriptome data to modify plant resistance in plant-microbe interactions, which is important for the study of growth and development and stress resistance of forest trees.

Since 2000, studies have analyzed the linkage of an *Alternaria* disease resistance gene in mandarin hybrids with RAPD fragments. Some studies on tomato genotypes for early blight disease resistance caused by *Alternaria* in Pakistan [25] found that *Alternaria* is very harmful to crops such as apple, citrus, tomato, mung bean and the study of *Alternaria* and its related resistance genes are important to improve the yield of economic forests and crops. At present, the study of this type of pathogen is still in a relatively superficial stage, it is necessary to continue to discover whether there exists a substance that can control the pathogenicity of *Alternaria* through continuous research. Exogenous sorbitol treatment has been shown to improve the disease resistance of apple fruit trees of the Rosaceae family against *Alternaria alternata* [16]. It relies on the method of plant genes affecting pathogen genes. Their study applied sorbitol to apple leaves and the *MdWRKY79* transcription factor responded to sorbitol and thus regulates the expression of *MdNLR16*. This is consistent with the view described in this study. In this study, we found that sorbitol feeding mainly by affecting *PsWRKY25*, which in turn affects the expression of the gene *PsCERK1*, generating metabolites that lead to certain defensive behaviors in the leaves. These findings suggest that sorbitol plays a signaling role in the regulation of plant disease resistance.

*WRKY* proteins often function as repressors and activators, members of the family which often plays roles in the repression and de-repression of important plant processes [26]. A single *WRKY* gene typically responds to multiple stressors, and their proteins may then act as negative or positive regulators involved in the regulation of a variety of seemingly disparate processes [27]. There are up to 100 such genes in Arabidopsis that are reasonably involved in various plant-specific physiological programs such as pathogen defense and senescence [28]. For example, *WRKY25* and *WRKY33* enhance Arabidopsis resistance to biotic and abiotic stresses such as NaCl and pathogens in Arabidopsis [29,30]. In addition to Arabidopsis, *WRKY* genes have disease resistance in crops such as rice (*Oryza sativa*), economic forest trees, play important roles in various developmental processes, including senescence and abiotic stresses [31,32]. Overexpression of the stress-induced gene *OsWRKY45* in Arabidopsis enhances disease resistance and confers drought tolerance [33]. Overexpression of the cotton (*Gossypium hirsutum*) *GhWRKY15* gene in tobacco (*Nicotiana tabacum NC89*) leads to increased resistance to viral and pathogen infections [34]. *WRKY* transcription factors often up-regulate the expression of several pathogen resistance-related genes by specifically binding to W-box elements in their promoter regions [35,36]. Overexpression of soybean *GmWRKY13* promotes lateral root development, this promotion may be the result of activation of the downstream gene *ARF6* [37]. Very closely linked to *WRKY* family genes are *CERK* kinases, *CERK* expression in plants activates the MARK pathway to improve disease resistance in Arabidopsis [38]. In the present study, same conclusion was reached. These studies corroborate the role of *WRKY* family genes in influencing plant responses to adversity and enhancing plant resistance to adversity. These adversities include abiotic adversities such as drought, salt stress and biotic adversities such as pathogen infestation. This illustrates that *WRKY* family genes are a key component of plant stress resistance research.

The plant-pathogen interaction was significantly enhanced in tea tree through the up-regulation of transcription factors such as *WRKY* and the synthesis of terpenoids [39]. Sorbitol feeding has a positive effect on resistance to apple leaf blight [16]. Suppression of sorbitol synthesis-related genes in apple leads to changes in the overall expression profile of stress response genes in leaves [40]. Sorbitol treatment of apples also suppressed Aspergillus in apple water kernel disease fruit [41]. Sorbitol not only has an important effect on plant disease resistance, but also has a specific role in plant resistance to abiotic stresses. For example, sorbitol improves potato resistance to water stress [42]. In this study, we found that overexpression of *PsWRKY25* in *Populus simonii* Carr. was accompanied by a changed expression of the corresponding *PsCERK1*, causing pathogen defense in plants. Overall, sorbitol is important for plant resistance to biotic and abiotic stresses, but relatively little research has been conducted on sorbitol as a signaling substance affecting disease resistance in poplar. Therefore, the study of sorbitol disease resistance in poplar is a very innovative work, which is an innovative point in the field of poplar disease resistance with high research value; the study of sorbitol’s role in these aspects is important for the study of plant stress resistance. 

## 5. Conclusions

In summary, sorbitol feeding was found to enhance *Populus simonii* Carr. disease resistance. The main reason is that after feeding, sorbitol acts as a signal substance, causing the up-regulation of the *PsWRKY25* in plants, which in turn affects the *PsCERK1*, thereby enhancing the susceptibility of the plants to pathogen infection and subsequently improving the disease resistance. This study provides a scientific basis for subsequent studies about sugar substance such as sorbitol, which is beneficial for the sustainable development of improvement of the resistance to disease in poplar and other forest trees. 

## Data Availability

The data supporting the study are presented in the supplementary data. Upon reasonable request, please consult the corresponding author if there are data not included in the supplementary data that you would like to know about.

## Supplementary Materials

The following are available online at https://www.mdpi.com/article/10.3390/genes13030405/s1, Figure S1: Statistics of DEGs, Figure S2: GO and KEGG enrichment analysis showed that DEGs between treatments, Figure S3: PCA analysis showing intra- sample and inter- sample correlation, Figure S4: The relative expression of *PsWRKY25* in different treatment periods, Table S1: Sequencing raw data statistics, Table S2: RT−qPCR primer sequences, Table S3: Vector primer sequences, Table S4: GO enrichment data, Table S5: KEGG enrichment data, Table S6: Venn data, Table S7: TF binding sites data, Table S8: DEGs data.
