# Supplementary material for: Sorbitol Reduces Sensitivity to Alternaria by Promoting Ceramide Kinases (CERK) Expression through Transcription Factor Pswrky25 in Populus (Populus simonii Carr.)
Source: Genes (Basel). 2022 Feb 24;13(3):405. doi: 10.3390/genes13030405 (PMC8954735; doi:10.3390/genes13030405)
Supplement: Supplementary file 1 [file genes-13-00405-s001.zip › Supplementary materials.pdf]

Supplementary materials

Supplementary Figures:

(**a**)

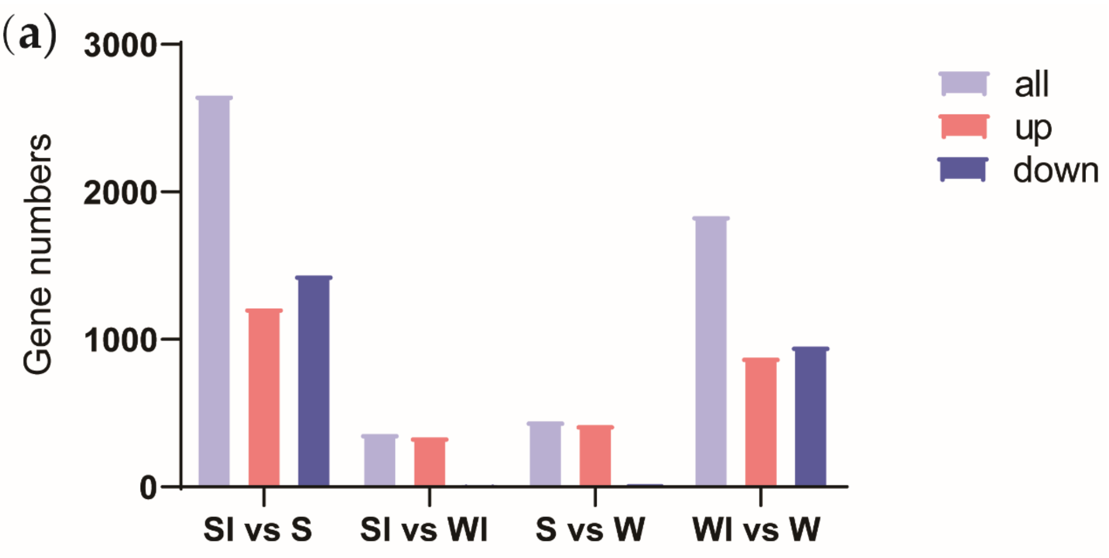

(**b**)

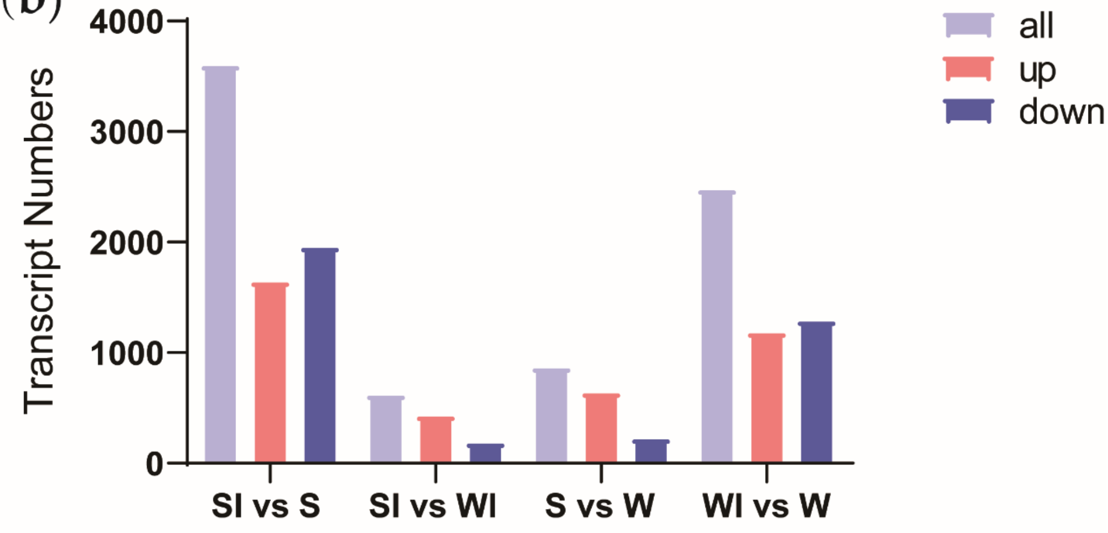

(c)

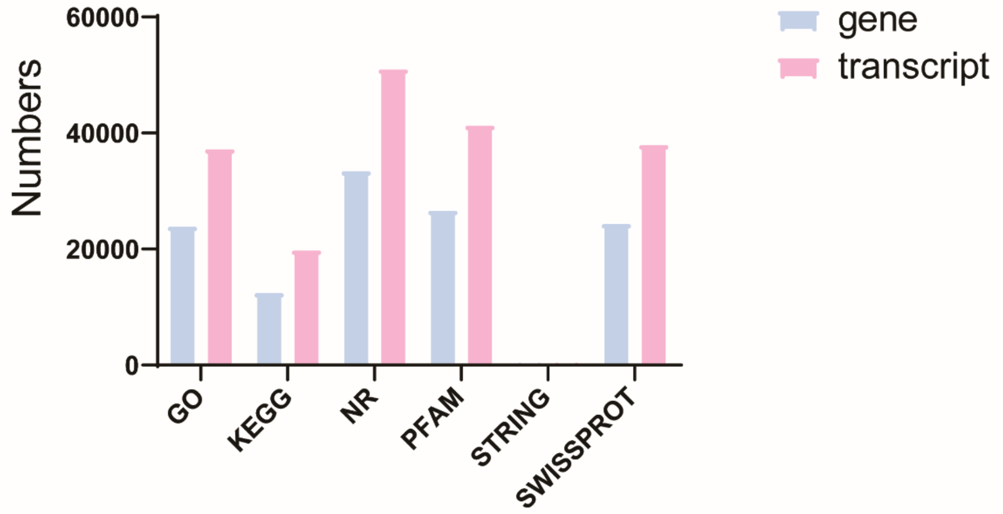

Figure S1: Statistics of DEGs.

(**a**) Statistics of all differential genes and the number of up-regulated and down-regulated differential genes in different groups. (**b**) Statistics of all differential transcription factors and up-regulated and down-regulated differential transcription factors in different groups. (**c**) Statistics of the number of differential genes in different annotation analysis. (W represents water feeding; WI represents water feeding then infestation; S represents sorbitol feeding; SI represents sorbitol feeding then infestation.).

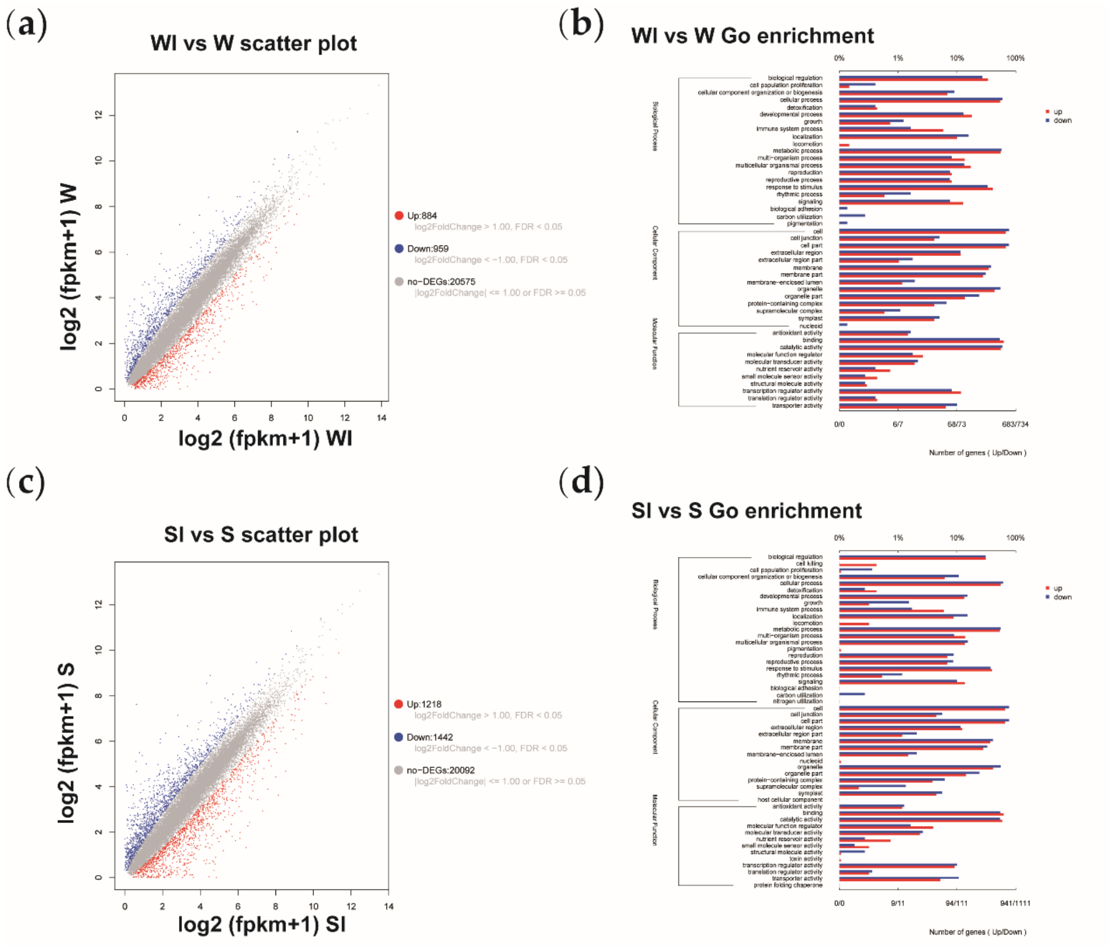

Figure S2: GO and KEGG enrichment analysis showed that differentially expressed genes between treatments.

(**a**) GO enrichment analysis of differential genes between WI and W. (**b**) KEGG enrichment analysis of differential genes between WI and W. (**c**) GO enrichment analysis of differential genes between SI and S. (**d**) KEGG enrichment analysis of differential gene between SI and S. (W represents water feeding; WI represents water feeding then infestation; S represents sorbitol feeding; SI represents sorbitol feeding then infestation.).

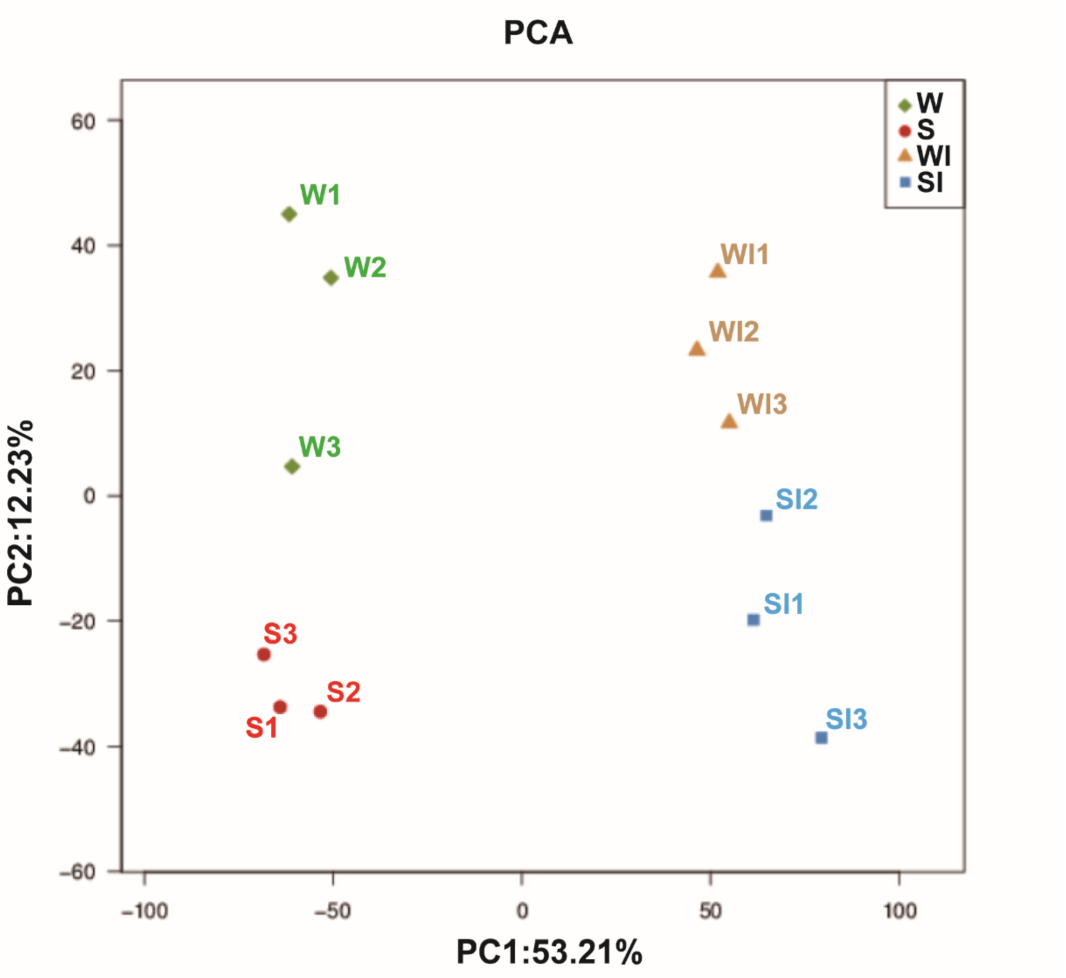

Figure S3: PCA analysis showing intra- sample and inter- sample correlation.

It shows the PCA analysis of 12 samples. (W represents water feeding; WI represents water feeding then infestation; S represents sorbitol feeding; SI represents sorbitol feeding then infestation. And the 1, 2, 3 represents three biological repetitions.).

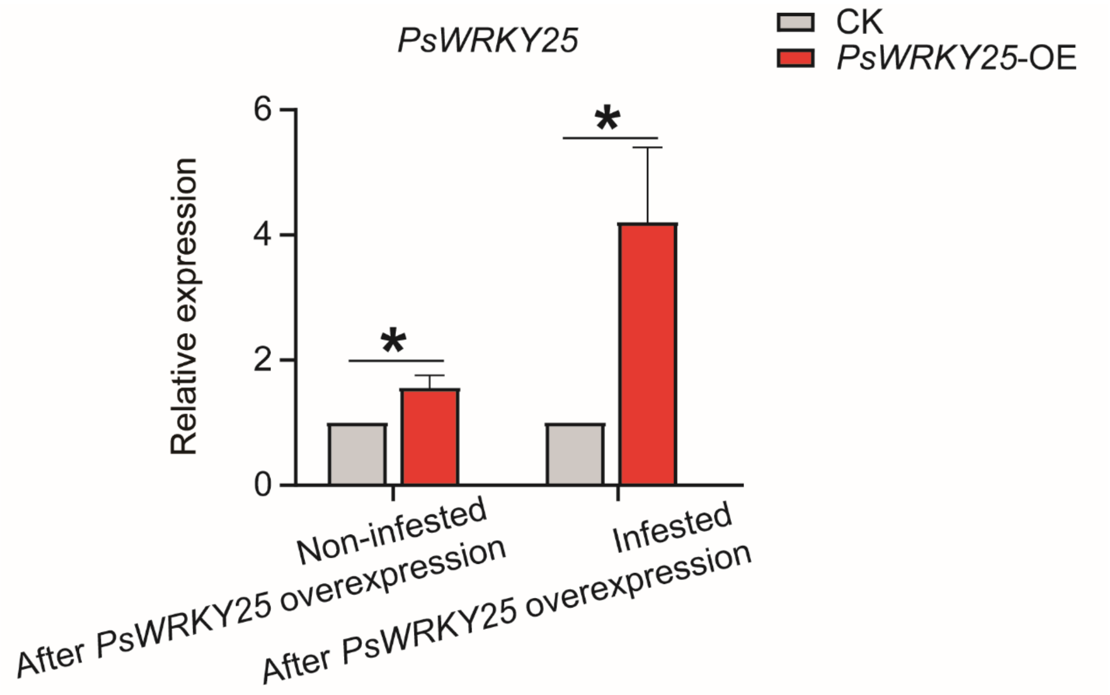

Figure S4: The relative expression of *PsWRKY25* in different treatment periods.

The relative expression of *PsWRKY25*. It was measured in leaves during different periods of pathogen infestation. (* stands for $p < 0.05$, with significant difference.).

Supplementary Tables:

Table S1: Sequencing raw data statistics.

Sample ID	Total Reads	Total Bases	Error%	Q20%	Q30%	GC%
WI3	43235154	6485273100	0.0284	96.44	91.37	45.46
WI2	56899720	8534958000	0.0279	96.71	91.7	44.1
WI1	43749624	6562443600	0.0286	96.33	91.21	45.73
W3	42014688	6302203200	0.0271	97.06	92.31	43.99
W2	45163140	6774471000	0.0281	96.64	91.57	44.09
W1	46431786	6964767900	0.028	96.69	91.59	45.12
SI3	48772064	7315809600	0.0277	96.77	91.84	45.37
SI2	53449888	8017483200	0.0273	96.95	92.17	44.23
SI1	48976330	7346449500	0.028	96.64	91.7	44.14
S3	67144730	10071709500	0.0279	96.7	91.66	45.48
S2	58828702	8824305300	0.0281	96.68	91.44	44.43
S1	48403090	7260463500	0.0281	96.58	91.57	44.12

Table S2: RT-qPCR primer sequences.

Primer	(5' to 3')	Base
UBQ-qPCR-F	AGACCTACACCAAGCCCAAGAAGAT	25
UBQ-qPCR-R	CCAGCACCGCACTCAGCATTAG	22
PtPR1-like1-qPCR-F	TTTGGCGTAATTCTGCTCGC	20
PtPR1-like1-qPCR-R	TGCCGGGTGGATCATAGTTG	20
PtPR1-like2-qPCR-F	CCACTAACCTGGGACACCAC	20
PtPR1-like2-qPCR-R	CCATGCAAGGTTCTCCCCAT	20
PtCML-qPCR-F	CGGGTGACTGGTCCGATATG	20
PtCML-qPCR-R	TTTCTCGAGACAAGGCCGTC	20
PtCPK-qPCR-F	ACTTGTCAGGGCAGCCAAAT	20
PtCPK-qPCR-R	CCCCTGCACAAAGCTCCATA	20
PtCPK26-qPCR-F	CCTCCAATTCCAAGCGCAAC	20
PtCPK26-qPCR-R	GCTGTCCTTCTTCGGGTTGA	20
PtCEPK1-qPCR-F	TACTCAGGAGTTGCTGCAGC	20
PtCEPK1-qPCR-R	CTTCAAGGGCCGGTAGCTAC	20
PtWRKY25-qPCR-F	ACCACAGCAACAATGGCAAC	20
PtWRKY25-qPCR-R	CGGATGTTGCTGTAGGTGGT	20
PtWRKY33-qPCR-F	GGACAACCTTAGCTGGGGAC	20
PtWRKY33-qPCR-R	GGGGGAGGAGAAAAGGGTTG	20

Table S3: Vector primer sequences.

Primer	(5' to 3')	Base
PtWRKY25-F	ATGGCTGCTTCTTCAGGGAG	20
PtWRKY25-R	CTACCAAAAACTCTCTACTTCCA	23

Table S4: GO enrichment data.

Due to the large amount of data, please see the "Supplementary Table ".

Table S5: KEGG enrichment data.

Due to the large amount of data, please see the "Supplementary Table ".

Table S6: Venn data.

Due to the large amount of data, please see the "Supplementary Table ".

Table S7: TF binding sites data.

Due to the large amount of data, please see the "Supplementary Table ".

Table S8: DEGs data.

Due to the large amount of data, please see the "Supplementary Table ".
